# Congenital Syphilis in South Africa: A Narrative Review of Epidemiology, Health System Drivers, Prevention, and the Path to Elimination

**DOI:** 10.3390/healthcare14213735

**Published:** 2026-11-03

**Authors:** Sibongile Phiri, Amanda Skepu, Zandile Nxumalo, Mosa Julia Letsielo, Victoria Oyekunle

**Affiliations:** 1 Human Diagnostic Platform, Council for Scientific and Industrial Research, Pretoria 0184, South Africa; askepu@csir.co.za (A.S.); znxumalo@csir.co.za (Z.N.); 2 Department of Agriculture and Animal Health, School of Agriculture and Life Sciences, College of Agriculture And Environmental Sciences, UNISA Science Campus, University of South Africa (UNISA), Johannesburg 1709, South Africa; 3 School of Health Systems and Public Health (SHSPH), Faculty of Health Sciences, Prinshof Campus (Health Sciences Campus), University of Pretoria, Pretoria 0002, South Africa; u14395691@tuks.co.za (M.J.L.); oluwatoyin.oyekunle@up.ac.za (V.O.)

**Keywords:** congenital syphilis, South Africa, *Treponema pallidum*, elimination, antenatal care, maternal health, vertical transmission, HIV, public health, surveillance

## Abstract

Congenital syphilis (CS), caused by vertical transmission of *Treponema pallidum* subsp. *pallidum*, remains a major but preventable cause of foetal and neonatal morbidity and mortality globally. Despite established antenatal screening programmes and the availability of effective treatment with benzathine penicillin G (BPG), South Africa continues to experience a substantial burden of maternal and congenital syphilis, with a 2023 national case rate approximately four times the World Health Organization (WHO) elimination target. This narrative review synthesises evidence from peer-reviewed publications, national surveillance reports, and policy documents identified through systematic searches conducted up to September 2026 [searched through August 2026] on congenital syphilis in South Africa. It addresses the microbiology and pathogenesis, epidemiology, clinical burden, diagnostic approaches, health system determinants, and strategies for elimination. Persistent transmission is associated with documented gaps in antenatal booking, repeat screening, treatment completion and partner management, and with recurrent benzathine penicillin shortages; HIV co-infection, poverty and other social and structural factors are likely contributors, but direct evidence for them is more limited. Congenital syphilis remains associated with adverse pregnancy outcomes including miscarriage, stillbirth, prematurity, neonatal mortality, and long-term disability. The elimination of congenital syphilis in South Africa requires strengthening antenatal care coverage, universal and repeat syphilis screening, prompt and complete maternal treatment, partner services, enhanced surveillance systems, and closer integration with HIV programmes. Progress towards the country’s 2028 elimination target will depend on a coordinated health systems approach aligned with WHO triple-elimination validation criteria.

## 1. Introduction

### 1.1. Syphilis as a Global Public Health Problem

Syphilis is a chronic, systemic sexually transmitted infection caused by the spirochaete *Treponema pallidum* subsp. *pallidum* [1]. When left untreated during pregnancy, it results in one of the most efficiently transmissible and consequential maternal infections, capable of causing miscarriage, stillbirth, prematurity, low birth weight, neonatal death, and a broad spectrum of congenital disease in surviving infants [2]. Without treatment, vertical transmission occurs in an estimated 70–100% of pregnancies with primary or secondary syphilis, approximately 40% in early latent syphilis and about 10% in late latent syphilis [3,4]. Furthermore, the global burden of congenital syphilis (CS) remains far above established elimination thresholds.

World Health Organization (WHO) estimates indicate that the global CS case rate increased to approximately 523 cases per 100,000 live births in 2022, corresponding to roughly 700,000 cases [5], exceeding by more than ten times the elimination threshold of 50 cases per 100,000 live births [6]. The burden was considerably higher in the WHO African Region, with an estimated 1260 cases per 100,000 live births in 2022, rising to approximately 1720 cases per 100,000 live births in western and central Africa [5,6]. These updated estimates highlight the persistent public health burden of congenital syphilis (CS) in Africa and the need to strengthen antenatal screening, diagnostic capacity, and the provision of timely treatment. Consistent with this pattern, Gilmour and Walls previously reported that the WHO African Region accounted for approximately 62% of global congenital syphilis cases in 2016, corresponding to an estimated 404,000 cases or 1119 cases per 100,000 live births [7].

### 1.2. Congenital Syphilis: Scientific Background

#### 1.2.1. Microbiology

*Treponema pallidum* is a thin, tightly helical spirochaete (approximately 6–20 μm in length) with a Gram-negative-like cell envelope and marked motility mediated by periplasmic flagella (endoflagella). The organism is microaerophilic and cannot be routinely cultured in standard in vitro microbiological media. This limitation has historically constrained direct culture-based laboratory diagnosis and has necessitated a reliance on serological methods [8]. In its genome, *T. pallidum* has a small, reduced chromosome consistent with adaptation to an obligate parasitic niche in humans, characterised by restricted biosynthetic pathways and substantial dependence on host-derived nutrients [8].

#### 1.2.2. Pathogenesis

**Figure 1:**
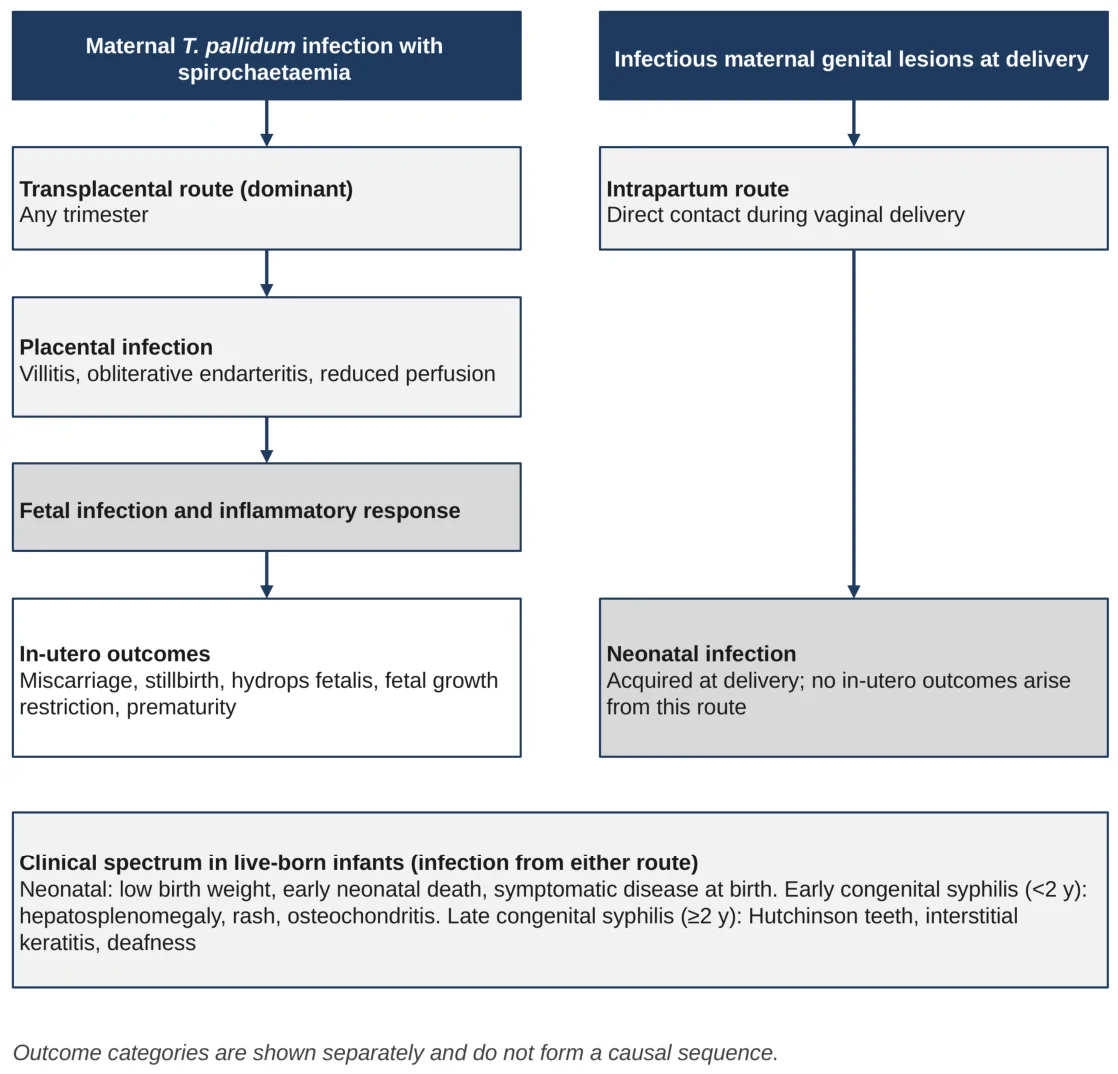
Transmission routes and outcomes of congenital syphilis. In the transplacental route (**left**), maternal spirochaetaemia leads to placental infection and foetal infection, which can cause in utero outcomes. In the intrapartum route (**right**), the neonate is infected by contact with maternal genital lesions at delivery, without placental involvement. Clinical spectrum categories are shown separately and do not form a causal sequence [2,7,10]. Note: HIV co-infection has been associated with an increased risk of adverse pregnancy outcomes [11,12].

Once the organism reaches the placenta via the pathways described in Section 1.3, placental infection and subsequent foetal invasion may occur. Recent molecular studies have begun to characterise the mechanisms underpinning these events. The treponemal surface protein Tp0954 has been identified as the first placental adhesin described to date and is capable of mediating attachment to trophoblast cells and extracellular matrix components, thereby providing a molecular basis for interaction with the placental barrier and subsequent tissue invasion [9]. Histopathological involvement of the placenta is associated with villitis, inflammatory plasma-cell infiltration and characteristic obliterative endarteritis of villous vessels, which impair placental perfusion [10]. These inflammatory and vasculopathic changes can disrupt the maternal–foetal exchange and contribute to placental dysfunction, foetal growth restriction and non-immune hydrops fetalis. After foetal invasion, *T. pallidum* can disseminate to multiple organ systems, including the liver, spleen, bone, skin and central nervous system, where infection induces both local and systemic inflammatory responses [10]. This results in the recognition of treponemal components by the innate immune system, thereby promoting inflammatory signalling and cytokine production that can contribute to tissue injury and the multisystem manifestations characteristic of congenital syphilis. Figure 1 summarises this sequence and its principal clinical consequences.

### 1.3. Vertical Transmission Mechanisms

The vertical transmission of syphilis occurs via two principal pathways. The first, and the dominant one, is transplacental transmission. Following maternal inoculation, *T. pallidum* disseminates haematogenously within days to weeks and can result in foetal infection at any gestational age, including the first trimester [2]. A second pathway is intrapartum transmission, whereby the neonate acquires infection through direct contact with active genital lesions during vaginal delivery. The mechanisms underlying placental invasion and foetal pathogenesis are described in Section 1.2.2 and Figure 1. In the absence of treatment, the risk of vertical transmission is strongly associated with the maternal stage of infection, reflecting higher circulating treponemal burden during early disease.

Transmission probability is highest in primary and secondary syphilis (about 70–100%), falls to about 40% in early latent syphilis, and declines further to about 10% in late latent syphilis [13]. Penicillin crosses the placenta and can prevent or treat foetal infection, and earlier treatment is more likely to prevent CS. Maternal treatment is considered adequate for neonatal risk classification when a stage-appropriate penicillin regimen was started at least 30 days before delivery and there is no evidence of re-infection or relapse [13]; treatment failure still occurs, particularly with late treatment, high maternal titres and re-infection [13]. HIV co-infection may further increase the risks associated with maternal syphilis. Pregnant women living with HIV who have syphilis co-infection experience an increased risk of adverse birth outcomes, including stillbirth and low birth weight. Moreover, maternal HIV–syphilis co-infection has been associated with an increased risk of mother-to-child HIV transmission compared with HIV infection alone, with Yeganeh et al. reporting approximately twofold higher adjusted odds of HIV transmission among co-infected women [11,12].

### 1.4. Why South Africa Matters

South Africa has provided routine antenatal syphilis screening since the late 1990s and has maintained a national prevention of mother-to-child transmission (PMTCT) programme for decades. Despite these long-standing interventions, congenital syphilis (CS) has re-emerged as a significant public health concern over the past decade. In response, South Africa classified CS as a Category 2 notifiable medical condition (NMC) in 2017, requiring notification to the National Institute for Communicable Diseases (NICD) within seven days [14]. In 2019, the national case definition was harmonised with the World Health Organization (WHO) criteria [14]. CS remains a pressing concern, particularly in settings with a high burden of sexually transmitted infections, including HIV [11]. Considering South Africa’s high antenatal HIV prevalence, marked socioeconomic inequality, and large public health system, South Africa is a critical setting in which to understand why a well-characterised, curable infection continues to cause preventable foetal and neonatal morbidity and mortality. This narrative review aims to synthesise the available evidence on the epidemiology, clinical burden, diagnosis, health system drivers and prevention of congenital syphilis (CS) in South Africa and to appraise the progress towards WHO elimination of mother-to-child transmission (EMTCT) of syphilis. Specifically, it addresses four questions: (1) What is the current burden and geographic distribution of maternal and congenital syphilis in South Africa? (2) How is CS diagnosed, and what are the limitations of current diagnostic approaches? (3) Which patient, provider, health system and social factors sustain transmission? (4) Which interventions are needed to close the gap for the WHO validation criteria?

## 2. Review Methodology

We conducted a narrative review of the literature on congenital syphilis (CS) and maternal syphilis in South Africa. PubMed/MEDLINE, Scopus, Web of Science, Google Scholar and SciELO were searched for literature published between 2000 and September 2026 using combinations of the following search terms: congenital syphilis, maternal syphilis, *Treponema pallidum*, syphilis in pregnancy, South Africa, antenatal care, screening, benzathine penicillin, HIV, surveillance and elimination. Grey literature was identified through searches of publications and resources from the National Institute for Communicable Diseases (NICD), South African National Department of Health (NDoH), South African National AIDS Council (SANAC) and World Health Organization (WHO). We included peer-reviewed studies, surveillance reports, clinical guidelines and policy documents addressing CS or maternal syphilis in South Africa. Relevant regional and international literature was also included to provide additional epidemiological and clinical context. We excluded studies with no direct relevance to South Africa, conference abstracts lacking sufficient methodological or outcome information, and publications in languages other than English. The retrieved literature was screened for relevance and eligibility, and the findings were synthesised thematically across epidemiology, pathogenesis, diagnosis, treatment, prevention, health system determinants, HIV co-infection and elimination strategies. No formal quality appraisal; risk-of-bias assessment or quantitative meta-analysis was undertaken.

## 3. Epidemiology of Congenital Syphilis in South Africa

Table 1 summarises the key congenital syphilis indicators globally and in South Africa, grouped by data source and level of evidence.

### 3.1. Global and Regional Epidemiology

The WHO estimated that about 700,000 cases of CS occurred globally in 2022, equivalent to a rate of 523 per 100,000 live births, more than ten times the elimination threshold of ≤50 per 100,000 [6,15]. This exceeded the 2020 estimate of 425 per 100,000, which was more than eight times the WHO elimination target [6] and the 2016 estimate of 473 per 100,000 [16]. The burden is unevenly distributed across WHO regions. In 2016, the African Region had by far the highest rate (1119 per 100,000 live births; 404,000 cases, representing about 62% of the global total), followed by the Eastern Mediterranean Region (635 per 100,000; about 17%) [7]. The corresponding estimate for 2012 was 469,000 cases, equivalent to 1377 per 100,000 live births [7,16]. Maternal syphilis prevalence in the region was estimated at 1.52% in 2016, more than twice the global average of 0.69%, with the region accounting for 57% of the global burden of syphilis in pregnancy [16]. Country-level modelling across 43 sub-Saharan African countries similarly estimated that maternal syphilis was responsible for more than 200,000 adverse pregnancy outcomes attributable to maternal syphilis annually, including approximately 60,000 cases of congenital syphilis, corresponding to an estimated 12.5 million disability-adjusted life-years, concentrated in the Democratic Republic of Congo, Nigeria, Ethiopia and Tanzania [17]. Substantial uncertainty persists, however, as few countries in the region report congenital syphilis case data to the WHO Global Health Observatory, and reported rates vary widely [7,16].

Regional syphilis prevalence among people living with HIV varies considerably across SSA, with pooled estimates around 7.3% overall and a lower pooled prevalence of approximately 3.1% reported for Southern Africa specifically, which highlights both the overlap between HIV and syphilis and the variation in prevalence across the region [18]. The pooled prevalence was 7.1% (95% CI 5.8–8.5%) in studies of people living with HIV conducted in antenatal care settings and 7.0% (95% CI 5.8–8.4%) among pregnant women living with HIV [18,19]. South Africa also reports a substantial burden, with a notified CS rate of 198 per 100,000 live births in 2023, about four times the WHO target of ≤50 per 100,000 (Section 3.2) [6]. This rate is based on clinically notified cases, so it cannot be compared directly with the WHO-modelled global and regional estimates above, which use different methods and include cases that are never diagnosed or reported. Differences in case definitions, diagnostic practice and reporting completeness also affect comparisons. Thus, the lower notified rate should therefore not be read as evidence that South Africa’s congenital syphilis burden is lower than the modelled estimates for the African Region.

### 3.2. South African Trends

Congenital syphilis has been notifiable in South Africa for decades under earlier communicable disease regulations [20], which gave the country a long-running, although historically incomplete, surveillance platform [21]. In 2017, it became a Category 2 notifiable medical condition (NMC), requiring healthcare workers to report diagnosed cases to the NICD within seven days, and its case definition was aligned with WHO surveillance criteria in 2019 [14]. Surveillance is supplemented by laboratory detection of rapid plasma reagin (RPR)-positive results among children younger than two years. These results are not equivalent to confirmed CS, because RPR positivity in young infants can reflect passively acquired maternal antibody, so they indicate trends rather than case counts. Laboratory surveillance of RPR seropositivity among children younger than two years showed rates of 89–127 per 100,000 live births in 2010–2018, rising to 165 in 2019, an increasing trend over the decade [22]. These are laboratory seropositivity rates, not notified cases. Notified clinical CS was 198 per 100,000 live births in 2023 (1739 cases, about four times the WHO elimination threshold) [14]. Improved ascertainment (a combined notification and investigation form introduced in July 2023) accounts for part of the recent increase [14]. In 2024, 1552 clinical CS notifications and 4754 RPR-positive results were recorded among children younger than two years [23]. These two sets of figures measure different things: the 2010–2019 rates are laboratory seropositivity rates, and the 2023–2024 rates are notified clinical cases. They therefore cannot be joined into a single trend.

### 3.3. Provincial Distribution

In 2023, four provinces (KwaZulu-Natal, Western Cape, Eastern Cape and Gauteng) accounted for 89.9% of the 1739 clinical notifications, and KwaZulu-Natal alone accounted for 46% [14]. Case rates per 100,000 live births were highest in the Western Cape (472) and KwaZulu-Natal (418), followed by the Eastern Cape (185), Mpumalanga (105), the Free State (85) and Gauteng (82). North West (65), the Northern Cape (49) and Limpopo (17) had the lowest rates, and only the Northern Cape and Limpopo were below the elimination threshold of 50 per 100,000 live births [14]. eThekwini recorded the highest district count (313 notifications) [14]. The four provinces with the most notifications also contributed 81% of RPR-positive results in children younger than two years, 73% of maternal syphilis cases in the 2022 antenatal sentinel survey and 66% of live births, which suggests that part of the variation reflects better detection and reporting in those provinces and that low rates elsewhere may reflect under-notification [14].

A facility-level audit at primary healthcare clinics in the Free State found inconsistent maternal syphilis management [24]. This illustrates gaps that may exist locally but does not explain provincial differences, which may also reflect differences in surveillance intensity and reporting completeness.

### 3.4. Maternal Syphilis Burden and Barriers to Care

#### 3.4.1. Maternal Syphilis Burden

In the 2019 national antenatal HIV sentinel survey, the weighted syphilis screening coverage among pregnant women attending antenatal care was 96.4%, which meets the WHO process target of 95%. Coverage was lower (93.5%) among HIV-positive women not yet established on antiretroviral therapy, which identifies a subgroup at higher risk of missed screening [25]. The same survey found that syphilis positivity was roughly double among HIV-positive women, regardless of antiretroviral therapy status [25]. Because the survey enrolled women who attended antenatal care, it does not capture women who never attend. A retrospective cohort study at midwife-run obstetric unit (MOU) Kwadabeka Community Health Centre (KCHC) in KwaZulu-Natal in 2018 reported an antenatal HIV prevalence of 44.3%, a syphilis prevalence of 3.8% and a syphilis incidence of 10.9 per 100 person-years, illustrating the scale of dual exposure that pregnant women face [26]. At the district level, antenatal syphilis prevalence is positively associated with HIV prevalence across South Africa (correlation coefficient 0.42), so the two epidemics overlap geographically as well as biologically [15]. This is an ecological association and does not show causation at the level of individual women.

#### 3.4.2. Barriers to Screening and Care

Qualitative and mixed-methods studies conducted in Johannesburg and Cape Town have described barriers affecting access to and the continuity of maternal health services among cross-border migrant and highly mobile women. Reported barriers include irregular legal status, language-related constraints, and interruptions in continuity of care when individuals traverse provincial and national borders, collectively undermining consistent engagement with antenatal care and prevention of mother-to-child transmission (PMTCT) services [27]. Foreign migrant adolescent girls appear to encounter additional, intersecting barriers related to both age- and migration-related barriers, including xenophobia and exclusion from youth-targeted services, which may reduce their visibility in routine antenatal care and surveillance systems [28]. As a result, women who initiate antenatal care late, as well as migrant/mobile populations, and adolescents are therefore likely to be under-represented in routine coverage statistics and may bear a disproportionate burden of undetected infection [27,28]. These observations come from qualitative or small studies and have not been quantified at the national level.

**Table 1:** Key congenital syphilis indicators globally and in South Africa, grouped by data source and level of evidence.

Indicator	Estimate	Level of Evidence	Reference
Global and regional (WHO-modelled estimates)			
Congenital syphilis (CS) rate, global	473 per 100,000 live births (2016); 425 (2020); 523 (2022; about 700,000 cases)	WHO-modelled estimate	[5,16]
CS rate, WHO African Region	1119 per 100,000 live births (404,000 cases, about 62% of global cases; 2016)	WHO-modelled estimate	[7]
Syphilis prevalence, sub-Saharan Africa (SSA)	Pooled 7.3% among people living with HIV (3.1% in Southern Africa); 7.1% (95% CI 5.8–8.5%) in studies conducted in antenatal care (ANC) settings; 7.0% (95% CI 5.8–8.4%) in pregnant women living with HIV	Meta-analysis	[18]
South Africa: National surveillance and surveys			
Notified clinical CS	198 per 100,000 live births (1739 cases; 2023); provincial range 17 (Limpopo) to 472 (Western Cape); KwaZulu-Natal 418. In 2024, 1552 cases	National surveillance (notified cases)	[14,21]
RPR seropositivity, children < 2 years	89–127 per 100,000 live births (2010–2018); 165 (2019)	National laboratory data (not notified cases)	[22]
Syphilis screening coverage, ANC	96.4% overall; 93.5% in HIV-positive women not on antiretroviral therapy (ART)	National antenatal sentinel survey, 2019	[25]
South Africa: Facility- and study-level data			
Antenatal HIV and syphilis	HIV prevalence 44.3%; syphilis prevalence 3.8%; syphilis incidence 10.9 per 100 person-years	Facility-based cohort	[26]
Mortality in symptomatic neonates with CS	26% (12 of 47)	Single-hospital case series, KwaZulu-Natal	[29]
Documented receipt of all three benzathine penicillin G (BPG) doses	About 36% of syphilis-positive women	Record review, Northern Cape and Gauteng (2013)	[30]
Targets			
WHO elimination threshold and South African commitment	≤50 CS cases per 100,000 live births; South Africa committed to elimination of mother-to-child transmission by 2028	Policy	[6,31]

Rates for different sources are not directly comparable: WHO figures are modelled estimates, whereas South African surveillance figures are notified or laboratory-detected cases. RPR, rapid plasma reagin; PLHIV, people living with HIV.

## 4. Clinical Burden and Outcomes

### 4.1. Foetal Outcomes

Untreated maternal syphilis is a major preventable cause of foetal loss. Syphilis-associated placental villitis and obliterative endarteritis impair uteroplacental perfusion, contributing to miscarriage, stillbirth, and foetal growth restriction [2,10]. In addition to these adverse pregnancy outcomes, severe foetal infection can result in systemic complications. Non-immune hydrops fetalis, characterised by generalised foetal oedema resulting from severe foetal anaemia, hepatic dysfunction, and cardiac compromise, represents a recognised and prognostically adverse manifestation of severe foetal infection and was identified as a factor independently associated with mortality in a South African neonatal case series [29].

### 4.2. Neonatal Outcomes

Among infants who survive to birth, congenital syphilis remains associated with an increased risk of prematurity, low birth weight, and early neonatal death. A retrospective review of the charts of 47 symptomatic newborns admitted with congenital syphilis to a peri-urban regional hospital in KwaZulu-Natal (Prince Mshiyeni Memorial Hospital, eThekwini) between January 2018 and March 2021 found that 12 newborns (26%) died, half of them within 24 h of birth. Hydrops fetalis, seizures, low Apgar scores and the need for delivery room resuscitation were identified as factors associated with mortality [29]. Similar severity has been documented in other South African settings. A tertiary neonatal unit in Cape Town described substantial morbidity among maternal syphilis management in a KwaZulu-Natal regional hospital, documenting a comparable cascade loss contributing to adverse neonatal outcomes [32].

### 4.3. Early Congenital Syphilis

Early congenital syphilis manifests within the first two years of life, most commonly in the neonatal period, and typically reflects an active and disseminated infection. Characteristic clinical manifestations include hepatosplenomegaly, maculopapular or bullous skin rash (including palms and soles), mucocutaneous lesions, condylomata lata, rhinitis (“snuffles”), generalised lymphadenopathy, osteochondritis, and periostitis of the long bones (visible radiographically and clinically as pseudoparalysis). Additional haematological and hepatobiliary findings include haemolytic anaemia, thrombocytopenia, and jaundice [13]. However, many infants with serological or other laboratory evidence of infection are asymptomatic at birth, which underscores the importance of serological screening rather than reliance on clinical detection alone.

### 4.4. Late Congenital Syphilis

Late congenital syphilis reflects the consequences of delayed clinical sequelae of in utero infection with *T. pallidum* that was untreated or inadequately treated during infancy, with clinical signs and symptoms typically emerging after two years of age. The classic triad described in the literature comprises Hutchinson teeth (notched, peg-shaped upper central incisors), interstitial keratitis (progressive corneal inflammation that can threaten vision), and sensorineural deafness, alongside other stigmata such as saddle-nose deformity, frontal bossing, sabre shins, and Clutton joints (symmetrical, painless knee effusions) [13]. Collectively, these largely irreversible sequelae highlight that optimal clinical management and public health strategy should prioritise prevention, timely diagnosis and early treatment, as late-stage morbidity cannot be fully reversed once established.

## 5. Diagnostic Approaches and Biomarkers

The laboratory tests used at each stage of the screening and diagnostic pathway, and their approximate performance characteristics, are summarized in Table 2.

### 5.1. Maternal Testing

Traditional screening relies on non-treponemal assays such as RPR and VDRL, which detect reagin antibodies and provide a quantitative titre for treatment monitoring, while treponemal assays (TPPA/TPHA, FTA-ABS, EIA/CLIA) serve as confirmatory tests or as initial screen in the reverse algorithm (Table 2).

#### 5.1.1. Non-Treponemal Tests (RPR, VDRL)

Non-treponemal tests (rapid plasma reagin (RPR) and Venereal Disease Research Laboratory (VDRL) use a cardiolipin–lecithin–cholesterol antigen and detect reagin antibodies, immunoglobulin G (IgG) and immunoglobulin M (IgM) produced in response to lipid material released from damaged host cells and from the treponeme. They therefore detect an antibody response rather than the organism [8,22]. These assays are inexpensive, rapid, and readily quantifiable as titres, which makes them well suited to both initial screening and, critically, to monitoring treatment response. Pooled estimates suggest an RPR sensitivity of approximately 86% and specificity of approximately 94%, though performance varies materially by disease stage, being lower in early primary infection and in some late latent cases. After adequate treatment, non-treponemal titres are generally expected to decline fourfold; however, the magnitude and timing of this decline vary according to the stage of infection, baseline titre, and history of previous infection. In pregnancy, a fourfold decline may not occur before delivery despite adequate treatment. Follow-up titres should generally not be repeated earlier than about 8 weeks after treatment unless re-infection or treatment failure is suspected, and the timing depends on the gestational age at treatment. In pregnancy, titres are usually repeated at 28–32 weeks of gestation and at delivery [36,37], and serological response should be interpreted together with the treatment history [36,38].

#### 5.1.2. Treponemal Tests (TPPA, TPHA, FTA-ABS, EIA, CLIA)

Treponemal tests, including *T. pallidum* particle agglutination (TPPA), haemagglutination (TPHA), fluorescent treponemal antibody absorption (FTA-ABS), and automated enzyme or chemiluminescence immunoassays (EIA/CLIA) detect antibodies specific to treponemal antigens. These tests are used to confirm a reactive non-treponemal screen (traditional algorithm) or as the initial high-throughput screen followed by non-treponemal confirmation (reverse algorithm) [5]. The reported sensitivity of FTA-ABS is reported at approximately 84% in primary infection, rising to near 100% in later stages, with specificity around 96%. Automated EIA/CLIA platforms typically demonstrate sensitivities around 99% with specificity in the 83–99% range depending on assay and population prevalence [33]. A key limitation of all treponemal tests is that, once reactive, they typically remain reactive for life regardless of treatment. As a result, they cannot reliably distinguish active from previously treated infection and are not used to monitor treatment response [8,22]. Neither the traditional nor the reverse algorithm is inherently superior; selection depends on local disease prevalence, laboratory throughput, and cost. A recent comparative study involving 26,519 pregnant women found similar proportions of confirmed syphilis with the traditional and reverse algorithms (0.2% vs. 0.3%), although the reverse algorithm resulted in a higher proportion of initially reactive screens (1.6% vs. 0.6%) and false-positive results [39]. These findings indicate that, although the two algorithms may identify comparable proportions of confirmed infection, the sequence of testing and subsequent reflex testing can substantially influence the number of women requiring further diagnostic evaluation [39]. Therefore, algorithm selection should be guided by the clinical and programmatic context rather than by an assumption that one approach is universally superior [40]. Importantly, evidence indicates that the choice and implementation of the testing algorithm can have direct consequences for clinical management and healthcare resource utilisation. Treponemal-only testing without confirmatory follow-up was associated with a three-fold overtreatment rate in a Zambian antenatal cohort [41]. In a maternity hospital in the Central African Republic, a modified reverse-testing algorithm reduced antibiotic and hospitalisation days for exposed newborns by 52% [42], which demonstrates that algorithm design directly changes neonatal resource use and outcomes in a resource-constrained setting comparable to South Africa.

### 5.2. Neonatal Diagnosis

**Figure 2:**
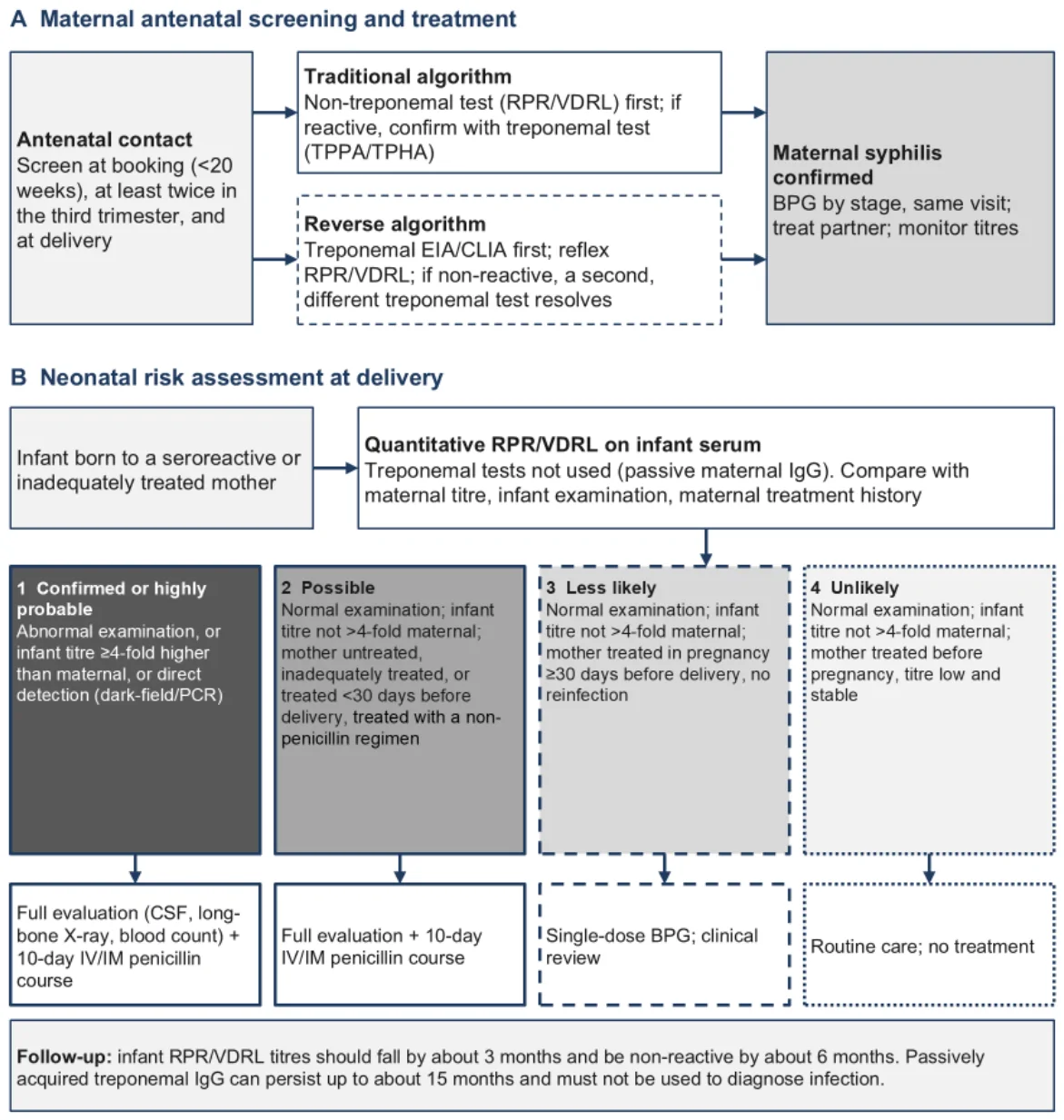
Maternal screening, treatment, and neonatal risk assessment, management and follow-up for syphilis in pregnancy [8,32,40,42].

Diagnosis of congenital syphilis in the neonate is complicated by passive transplacental transfer of maternal IgG, which can persist in infant serum for up to about 15 months whether or not the infant is infected (Table 2) [33]. Thus, treponemal tests (TPPA, FTA-ABS, EIA, and CLIA) on neonatal serum are therefore not recommended for diagnosis, because a reactive result may reflect maternal antibody rather than foetal infection [33]. Diagnosis instead relies on a quantitative non-treponemal test (RPR or VDRL) on infant serum, interpreted with the maternal titre, the physical examination, the adequacy of maternal treatment and, where available, placental histopathology or long-bone radiography. The US CDC formalises this in four risk categories [33,36]. Confirmed or highly probable CS applies to an infant with an abnormal examination, a non-treponemal titre at least fourfold higher than the maternal titre, or direct detection of T. pallidum. Possible CS applies to an infant with a normal examination and a titre not more than fourfold higher than the maternal titre, whose mother was untreated, inadequately treated, treated with a non-penicillin regimen, or treated less than 30 days before delivery. Less likely CS applies to an infant with a normal examination and a titre not more than fourfold higher than the maternal titre, whose mother received a stage-appropriate penicillin regimen started at least 30 days before delivery, with no evidence of re-infection or relapse. Unlikely CS applies to an infant with a normal examination and a titre not more than fourfold higher than the maternal titre, whose mother was adequately treated before pregnancy and whose titre remained low and stable during pregnancy and at delivery. A South African study found that a maternal VDRL titre of at least 1:32 predicted congenital syphilis with 93% sensitivity and 78% specificity, a threshold that can help triage where full neonatal evaluation is not immediately feasible [34]. Figure 2 summarises the maternal and neonatal algorithm.

### 5.3. Novel Biomarkers

Direct molecular detection of *T. pallidum* DNA by polymerase chain reaction (PCR) or other nucleic acid amplification tests (NAATs) on lesion exudate, placental tissue, or neonatal specimens offers high specificity and can support diagnosis in serologically equivocal cases, though availability remains largely confined to reference and research laboratories rather than routine antenatal or neonatal care in South Africa [35]. Placental pathology, particularly the presence of villitis, obliterative endarteritis, and villous immaturity, can provide supporting evidence of congenital infection when samples are examined [10]. Emerging point-of-care approaches that assess the treponemal IgM signal in neonatal samples have shown promising discrimination between risk categories in early studies, correlating with the established CS risk stratification of CS, though such assays remain investigational and are not yet part of routine algorithms [33].

## 6. Drivers of Persistent and Rising Transmission

### 6.1. Patient-Level Factors

Late or absent antenatal booking is the first and largest gap in the South African prevention cascade. The initiation of antenatal care after 20 weeks of gestation shortens the window available for screening, treatment, and confirmation of adequate response before delivery. Among the 938 cases of congenital syphilis reported nationally between January 2020 and June 2022, only 57% of mothers had an antenatal booking visit at all, and the median interval between the first syphilis test and delivery was just 16 days overall, rising to 82 days among women who did book, which leaves too little time for screening, treatment, and confirmation of response before delivery [43]. This is not a new problem. Studies conducted in South Africa in the late 1990s documented the same pattern of late presentation and measurable impact on perinatal mortality from missed treatment opportunities in rural settings. Poor health-seeking behaviour, shaped by stigma associated with sexually transmitted infection, low perceived personal risk, transport and distance barriers to facilities, and competing household demands, further delays first-contact antenatal services in many South African settings [24]. A prospective study across 12 antenatal clinics in Hlabisa, KwaZulu-Natal, found a mean gestation of 24 weeks at the first antenatal visit—and 19% of women diagnosed with syphilis received no treatment at all—and 11% perinatal mortality among those diagnosed [43]; a companion case-control and cohort study in the same district found an adjusted odds ratio of 11.8 for an adverse pregnancy outcome among syphilis-positive women [43]. Together, these indicate that late booking and incomplete treatment have been a persistent, rather than emerging, driver for decades.

### 6.2. Provider-Level Factors

Missed screening opportunities occur when repeat testing is not offered at subsequent antenatal visits or in labour and delivery for women with no or late antenatal contact. Even when infection is detected, the start of treatment can be delayed by clinic workflow, and atypical or subtle clinical presentations of maternal or congenital disease may go unrecognised by staff without regular refresher training, which contributes to both missed treatment and incomplete case notification [14]. Facility-level failures are concentrated on the treatment-completion step rather than the initial testing step. Evidence from records reviews and audits indicates substantial attrition between diagnosis and completion of recommended therapy. A record review in the Northern Cape and Gauteng found that only 36% of syphilis-positive women had documented receipt of all three recommended benzathine penicillin injections [30]. In Free State primary healthcare facilities, 44.7% of syphilis-positive patients were never retested after their initial positive result, which precluded confirmation of adequate serological response [24]. A clinical audit of maternal syphilis management in a KwaZulu-Natal regional hospital quantified comparable cascade losses between diagnosis and completed treatment [27]. Furthermore, because CS is a notifiable medical condition, the availability of various methods for reporting a notifiable medical condition—namely, the Notifiable Medical Conditions Electronic Application, a web-based platform and a paper-based notification through the use of both the Case Notification Form and a Case investigation form—may influence the observed reporting trends of the case notifications reported [23]. The selection of a notification method by a healthcare provider may have an impact on timely reporting, as electronic notification methods assist in facilitating faster and timely case notifications, in contrast to paper-based notification methods, which may lead to delays [14].

### 6.3. Health System Drivers

Benzathine penicillin G (BPG) is the only treatment recommended by international guidelines for syphilis in pregnancy, because no adequately studied alternative reliably crosses the placenta to treat foetal infection [9]. BPG is an older, off-patent, low-margin injectable medicine produced by a narrow global manufacturing base, which makes it vulnerable to recurrent international shortages; a multi-country survey found that 41% of responding countries and territories had experienced a BPG shortage over a two-year period [44,45]. South Africa has been among the countries affected, which has prompted the national Department of Health to issue interim guidance on therapeutic alternatives. Similar stock-outs have been documented in other high-burden African settings such as Zambia, where supply interruptions were identified as a major barrier to documented syphilis treatment in pregnancy [44]. A broader regional review found similar implementation gaps in antenatal syphilis detection and treatment across health facilities in sub-Saharan Africa [46], with modelling suggesting a substantial public health burden of adverse pregnancy outcomes attributable to syphilis across 43 sub-Saharan African countries [17]. These supply constraints are further exacerbated by human resource shortages, high antenatal clinic workloads, and fragmented laboratory turnaround times, and weak defaulter-tracing and referral systems compound these supply-side constraints.

### 6.4. Social Determinants

Social and structural factors shape both exposure to syphilis and access to antenatal care. The strongest South African evidence concerns access. Among 938 mothers of congenital syphilis cases notified between January 2020 and June 2022, only 57% had booked antenatal care, and the median interval between the first syphilis test and delivery was 16 days [47]. Young maternal age, unplanned pregnancy and late recognition of pregnancy have been linked to delayed booking in South African surveillance [47]. Studies in Soweto and at an urban community health centre in Gauteng identified pregnancy unawareness, transport costs, distance to facilities and competing household or work responsibilities as reasons for late antenatal care [48,49], and stigma and transport barriers have been described in Free State primary care [24]. Cross-border migrant and highly mobile women in Johannesburg and Cape Town face undocumented status, language barriers and disrupted continuity of care, which affect consistent antenatal and PMTCT care [27].

Foreign migrant and highly mobile women and adolescent girls face additional barriers like xenophobia and exclusion from youth-targeted services, which reduces their contact with routine antenatal care [28]. These groups are likely to be under-represented in routine screening statistics. These factors act mainly by delaying or interrupting access to screening, treatment and follow-up, not as direct causes of infection. Poverty, food insecurity, gender inequality, gender-based violence and informal settlements are plausible contributors, but studies linking them directly to maternal or congenital syphilis in South Africa are lacking.

### 6.5. HIV–Syphilis Syndemic

**Figure 3:**
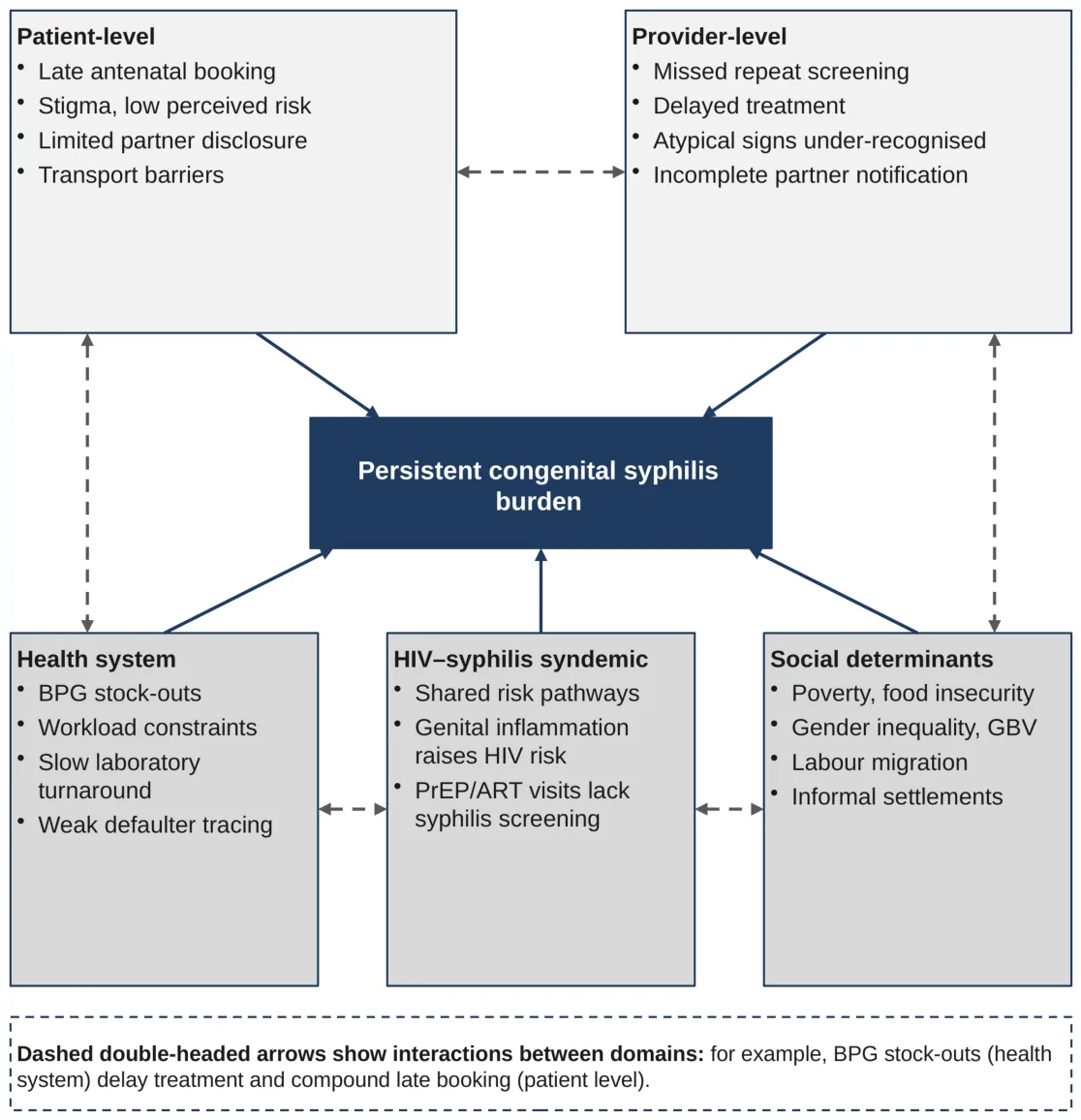
Multi-level drivers of persistent congenital syphilis in South Africa [18,24,30,45,46,47,52].

We use ‘syndemic’ to describe co-occurring epidemics that cluster in the same populations and interact through shared social and structural drivers. Epidemiological overlap is shown by the positive association between antenatal HIV and syphilis prevalence across South African districts [15]. Biological interaction is also described: genital ulceration and inflammation from syphilis increase HIV acquisition and shedding [50], and maternal HIV–syphilis co-infection has been associated with a higher risk of HIV transmission to the infant than HIV infection alone [18]. HIV can occasionally alter the serological response to syphilis, but standard serological tests remain reliable in most people with HIV [51]. Pregnant women living with HIV also face an elevated risk of syphilis co-infection, which reflects shared behavioural risk pathways [18]. In the 2019 national antenatal sentinel survey, syphilis positivity was roughly double among HIV-positive women regardless of antiretroviral therapy status, and antenatal syphilis prevalence remains positively correlated with HIV prevalence across South African districts [25,26]. South Africa’s large and growing antenatal pre-exposure prophylaxis (PrEP) programme has highlighted a further integration gap: an analysis of antenatal PrEP services found that pregnant women receiving PrEP, a population at elevated behavioural and biological risk of both HIV and syphilis, were not being systematically monitored for syphilis outcomes, representing a missed opportunity within an existing point of contact with the health system [46,52]. Figure 3 synthesises these interacting patient-level, provider-level, health system, social, and syndemic drivers.

## 7. Prevention, Treatment and the Elimination Agenda

### 7.1. Prevention Strategies

The prevention of congenital syphilis in South Africa requires a combination of repeated antenatal screening, accessible POC testing, prompt treatment, and effective partner management. The NDoH recommends repeated syphilis screening during pregnancy, with the 2023 national guidance strengthening the integration of HIV and syphilis testing through dual rapid diagnostic tests [14]. The 2023 guideline specifies at least four syphilis tests in pregnancy: at booking (before 20 weeks), at least twice in the third trimester, and at delivery [14]. Repeated testing is important because women may acquire syphilis after an initial negative antenatal test, while early identification provides an opportunity for timely treatment before delivery. POC rapid diagnostic tests can further strengthen this prevention pathway by enabling testing and treatment during the same antenatal visit, reducing dependence on laboratory turnaround times and return visits for results [30,53]. This approach is particularly relevant in settings where women may experience difficulties returning to health facilities because of geographical, transport, or socioeconomic barriers. Integration of dual HIV/syphilis testing may additionally simplify antenatal screening by allowing both infections to be assessed during a single healthcare encounter [54]. Partner identification, notification, testing, and treatment are also essential components of prevention because untreated partners can result in maternal re-infection during pregnancy and compromise the effectiveness of an initially adequate treatment course. In South Africa, strengthening partner management remains important for closing gaps in the syphilis prevention cascade. Collectively, these strategies support a prevention approach that moves beyond single-point screening towards repeated testing, same-day diagnosis and treatment, and prevention of re-infection, thereby increasing the likelihood that maternal syphilis is identified and adequately managed before delivery [14].

### 7.2. Treatment

#### 7.2.1. Benzathine Penicillin G—Mechanism of Action

Benzathine penicillin G (BPG) is a β-lactam antimicrobial agent whose active component, benzylpenicillin (penicillin G), inhibits penicillin-binding proteins (PBPs), including D, D-transpeptidases, thereby blocking the final transpeptidation step of peptidoglycan synthesis and compromising bacterial cell-wall integrity [55,56]. Because the benzathine formulation is slowly absorbed from the intramuscular injection site, it provides prolonged systemic exposure to penicillin G [56]. Pharmacokinetic evidence in pregnant women with syphilis demonstrated that a three-dose, once-weekly BPG regimen maintained benzylpenicillin concentrations above the target threshold for at least four weeks, supporting sustained treponemicidal exposure during pregnancy [56]. The clinical regimen should, however, remain aligned with the stage of syphilis and current treatment guidelines. Recent evidence comparing single- and three-dose BPG regimens for early syphilis in pregnancy found no significant difference in maternal serological response or neonatal outcomes, supporting the current single-dose regimen for early syphilis [57].

#### 7.2.2. Maternal and Neonatal Treatment

The standard regimen is a single 2.4-million-unit intramuscular dose of benzathine penicillin G (BPG) for early-stage disease (primary, secondary, or early latent syphilis) or three weekly doses of 2.4 million units for late latent or unknown-duration infection [13]. For the purposes of neonatal risk stratification, maternal treatment is considered adequate when a stage-appropriate regimen was started at least 30 days before delivery and there is no evidence of re-infection or relapse. Infants classified as having confirmed, highly probable, or possible congenital syphilis require appropriate diagnostic evaluation and a 10-day course of intravenous aqueous crystalline penicillin G or intramuscular procaine penicillin G, whereas infants at lower risk may be managed with a single dose of BPG and appropriate clinical and serological follow-up, as summarised in Figure 2 [21,26,33]. Importantly, no confirmed clinically significant resistance of *Treponema pallidum* to penicillin has been documented, although resistance to some alternative antimicrobials has emerged [13]. Consequently, persistent infection or congenital syphilis following maternal treatment is more appropriately considered in relation to factors such as delayed initiation, incomplete treatment, inadequate timing before delivery, or re-infection rather than established penicillin resistance. Ensuring timely diagnosis, completion of the recommended treatment regimen, and prevention of re-infection therefore remain central to effective maternal and congenital syphilis prevention.

During BPG shortages, available stock should be prioritised for pregnant women and infants. In 2018, during a national shortage, the National Department of Health prioritised pregnant women for BPG, while other patients were treated with doxycycline [14]. Alternative antibiotics have limited evidence in pregnancy and do not reliably treat the foetus, so they should be used only under specialist guidance, and exposed infants should be evaluated as if maternal treatment had been inadequate [13,45].

### 7.3. WHO Elimination Targets

**Figure 4:**
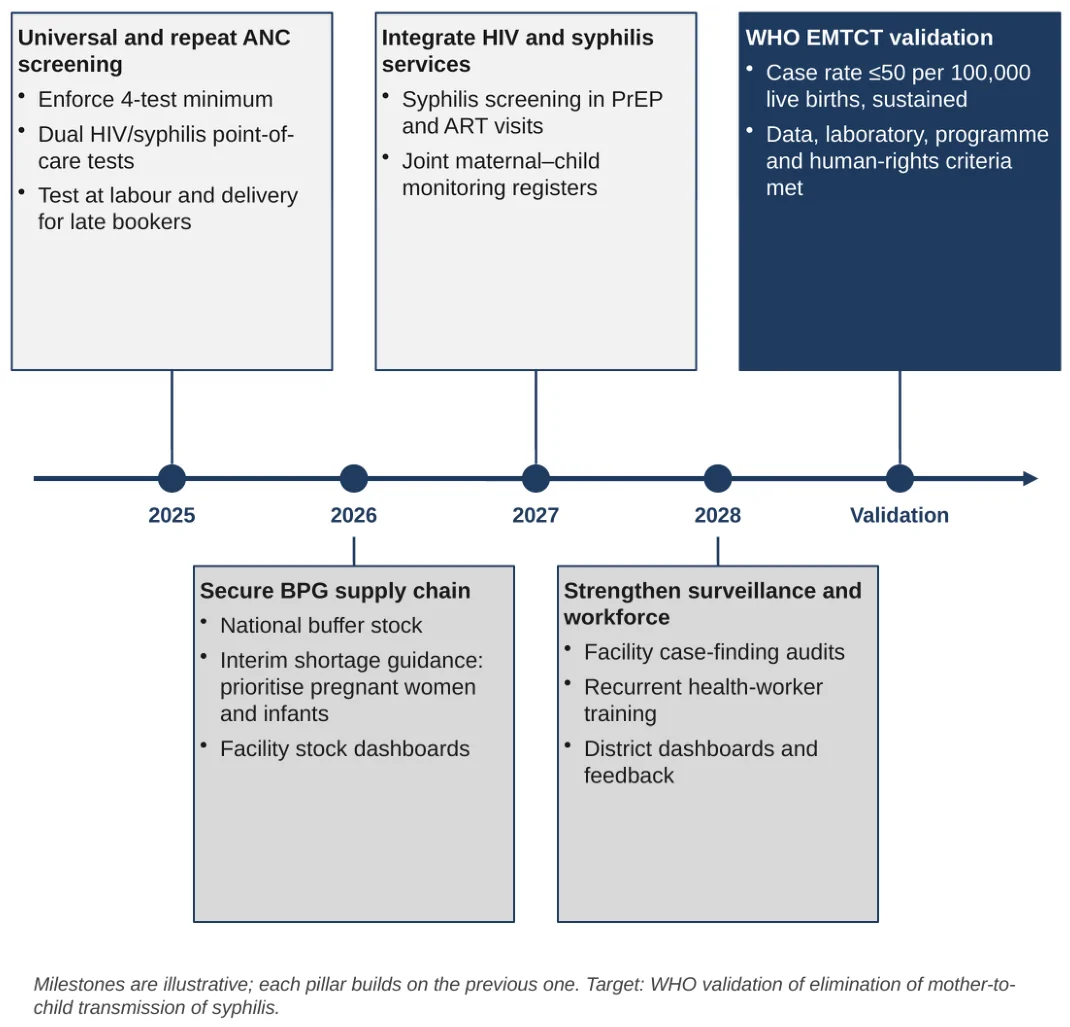
Roadmap to elimination of mother-to-child transmission of syphilis in South Africa aligned with the WHO triple-elimination (EMTCT) validation framework [14,58,31].

South Africa has committed to eliminating mother-to-child transmission (EMTCT) of syphilis by 2028, in line with its National Strategic Plan for HIV, TB and STIs 2023–2028 [2,22]. WHO’s validation framework defines EMTCT of syphilis through an impact threshold of ≤50 congenital syphilis cases per 100,000 live births, together with high coverage of antenatal care, syphilis testing and adequate treatment, supported by quality data and laboratory systems and respect for human rights, gender equality and community engagement [25,58]. For countries progressing towards elimination, WHO also provides a Path to Elimination framework with bronze, silver and gold levels of achievement [58]. The 2025 WHO country guidance further recommends an integrated, person-centred approach to the triple elimination of HIV, syphilis and hepatitis B, structured around four pillars: primary prevention of infection and vertical transmission; sexual and reproductive health linkages and integration; essential maternal EMTCT services; and infant, child and partner services [59]. For South Africa, this framework provides a basis for strengthening existing maternal and child health programmes through integrated service delivery, improved surveillance and laboratory systems, community engagement, and coordinated implementation towards the 2028 national commitment [31]. An illustrative sequence of the main actions, from universal and repeat screening to validation, is shown in Figure 4.

## 8. Discussion: Interpreting the South African Picture

### 8.1. Why Cases Persist Despite Available Solutions

Taken together, the available evidence suggests that the persistent burden of CS in South Africa reflects gaps across the prevention and care cascade rather than the lack of effective diagnostic or therapeutic tools [2]. The main interventions, such as screening for syphilis, point-of-care testing, and effective treatment with benzathine penicillin G, are established and available within the national health system, yet important gaps remain in timely access to antenatal care, repeated screening, completion of treatment, and follow-up. The coverage of syphilis screening among women attending antenatal care was high (96.4%) in the 2019 sentinel survey [25]; however, this figure does not capture women who do not attend antenatal care. Indeed, only 57% of mothers of reported congenital syphilis cases had documented antenatal care attendance [47]. Further gaps occur after diagnosis: studies have documented incomplete treatment and inadequate follow-up among women with syphilis [52]. These findings indicate that health system and implementation barriers are important contributors to persistent CS, while provider knowledge, under-recognition of infection, and gaps in diagnostic implementation may also contribute.

### 8.2. Comparison with Other LMICs

South Africa’s experience reflects challenges documented across other low- and middle-income countries, while international examples demonstrate different approaches to strengthening prevention and elimination. In Zambia, benzathine penicillin G (BPG) stock-outs were identified as an important barrier to timely maternal syphilis treatment, which highlights the importance of reliable commodity supply for South Africa [44]. In the Central African Republic, a modified reverse-testing algorithm reduced antibiotic use and hospitalisation days among exposed newborns, which illustrates how context-appropriate diagnostic strategies may improve the efficiency of congenital syphilis management in resource-constrained settings [42]. Regional progress also provides relevant implementation lessons. Botswana achieved WHO Gold Tier status on the Path to Elimination of mother-to-child transmission of HIV in 2025, following Silver Tier recognition in 2021, while Namibia achieved Bronze Tier status for HIV in 2023 and Silver Tier status for hepatitis B [58,59]. These achievements highlight the value of sustained political commitment, integrated services, improved primary healthcare, quality data systems and community engagement, rather than representing syphilis-specific achievements [58]. Beyond Africa, Cuba became the first country validated by WHO for elimination of mother-to-child transmission of both HIV and syphilis in 2015, followed by Thailand in 2016 [58]. These experiences demonstrate that sustained antenatal coverage, routine syphilis testing, timely treatment, integration of maternal and child health with sexual and reproductive health services, and strong surveillance systems can support elimination. For South Africa, the key lesson is the need to translate the availability of effective interventions into consistent implementation across the entire prevention cascade while adapting proven approaches to local epidemiological, health system and social contexts.

### 8.3. Lessons from HIV EMTCT Programmes

South Africa’s experience in preventing mother-to-child transmission (PMTCT) of HIV provides relevant lessons for congenital syphilis prevention because both programmes rely substantially on the antenatal care platform. The expansion of integrated HIV and syphilis testing, including dual point-of-care testing, provides an opportunity to increase screening efficiency at the same antenatal contact [58,59]. Other potentially transferable programme elements include clearly defined national targets, routine monitoring through health information systems and district-level performance review, strengthened procurement and buffer stock systems for essential medicines and diagnostics, community outreach and tracing of patients who miss care, and partner testing and treatment [58,59]. These approaches are consistent with the broader WHO triple-elimination framework, which emphasises integrated maternal services, reliable commodities, quality data, community engagement, and linkage to care [59]. However, not all components of HIV PMTCT are directly transferable. HIV prevention requires lifelong treatment and sustained retention in care, whereas syphilis is curable with antibiotic treatment but requires timely treatment, prevention of re-infection, repeat testing during pregnancy, and assessment of treatment response. South Africa’s substantial reduction in mother-to-child HIV transmission therefore illustrates the potential of sustained political commitment, integrated antenatal services, monitoring systems, and reliable commodity supply, while the continuing congenital syphilis burden highlights the need to apply these programme strengths to the specific diagnostic, treatment, follow-up, and re-infection-prevention requirements of syphilis [58,59].

### 8.4. Distance from WHO Validation Criteria and Priorities to Close the Gap

South Africa remains above the WHO congenital syphilis validation threshold. With a notified congenital syphilis rate of 198 per 100,000 live births in 2023 [14], approximately a 75% reduction would be required to reach the WHO impact threshold of ≤50 cases per 100,000 live births [58]. Although syphilis screening coverage among women attending antenatal care reached 96.4% in the 2019 sentinel survey [25], available evidence indicates that other components of the validation framework, including timely antenatal care, adequate maternal treatment and completeness of surveillance data, cannot yet be demonstrated consistently at the required levels [30,47]. Marked provincial variation in reported congenital syphilis rates, ranging from 17 to 472 cases per 100,000 live births, may reflect differences in epidemiology, health system performance, and surveillance intensity and should therefore not be interpreted as evidence of causal differences in provincial performance [14]. Closing the gap by 2028 for WHO validation will require sustained improvements across the prevention cascade, particularly in early antenatal engagement, timely and complete maternal treatment, prevention of re-infection, partner management, and robust surveillance and data systems. South Africa’s 2028 elimination commitment provides an important national target against which progress can be measured, while the WHO Path to Elimination framework offers an intermediate mechanism for recognising incremental progress towards eventual validation [58].

## 9. Recommendations

The recommendations below cover clinical, public health, policy and research priorities, and the main strategic actions are summarised by health-system level in Table 3.

### 9.1. Clinical Recommendations

To strengthen the early detection and management of maternal syphilis and reduce the risk of congenital syphilis, syphilis testing should be performed at least four times in pregnancy (at booking before 20 weeks, at least twice in the third trimester, and at delivery), particularly among women with no or late antenatal care, in accordance with the 2023 national guidelines [14]. In addition, healthcare providers should consistently apply the structured neonatal risk-classification algorithm (Figure 2) to guide appropriate treatment intensity, clinical management, and follow-up of neonates at risk of congenital syphilis [33].

### 9.2. Public Health Recommendations

Syphilis screening and follow-up should be integrated into existing maternal HIV services, including antenatal pre-exposure prophylaxis (PrEP) and antiretroviral therapy (ART) programmes, coupled with health educational interventions to improve knowledge on syphilis and address documented gaps in monitoring and continuity of care [52]. In parallel, partner notification and treatment pathways should be strengthened to ensure the timely identification and management of sexual partners, thereby reducing the risk of maternal re-infection and subsequent adverse pregnancy outcomes [24,30].

### 9.3. Policy Recommendations

A resilient supply of benzathine penicillin G (BPG) should be ensured through national buffer stocks, routine facility-level stock monitoring, early identification of supply disruptions, and clear national guidance for managing periods of shortage [13,45]. During BPG shortages, available stock should be prioritised for pregnant women with syphilis and for infants requiring treatment. Alternative antibiotics have limited evidence for the prevention of congenital syphilis in pregnancy and should not be considered equivalent substitutes for BPG; where BPG is unavailable, management should follow current South African Department of Health guidance and specialist advice, with appropriate evaluation and follow-up for exposed infants [13,45]. Geographic prioritisation should not be based on notification rates alone. Districts requiring intensified intervention should be identified using a combination of reported congenital syphilis and maternal syphilis burden, antenatal care and screening indicators, treatment initiation and completion, repeat testing, partner management, and assessments of surveillance completeness. This approach is important because low notification rates may reflect differences in case ascertainment, reporting completeness, or surveillance intensity rather than a genuinely lower disease burden. Targeted strengthening of screening, treatment, partner management, follow-up, commodity availability, and surveillance should therefore be directed towards areas where multiple indicators demonstrate gaps in the prevention and care cascade [14,58].

### 9.4. Research Recommendations

Facility-based case-finding audits should be conducted to assess the completeness of congenital syphilis surveillance and improve the accuracy of burden estimates, particularly in provinces reporting unexpectedly low notification rates [14,24]. In parallel, further research should evaluate the diagnostic performance and clinical utility of point-of-care molecular and IgM-based assays for their potential to improve neonatal risk stratification and inform timely management of congenital syphilis in South African healthcare settings [33,42].

## 10. Conclusions

Congenital syphilis remains a preventable but persistent problem in South Africa. The notified rate in 2023 (198 per 100,000 live births) is about four times the WHO threshold, with wide provincial variation and severe neonatal outcomes still reaching neonatal units [14,29]. Available tools and a well-established antenatal platform exist, but late booking, incomplete treatment, weak partner services and imperfect surveillance limit their impact, alongside diagnostic and provider-level gaps. Reaching WHO validation requires sustained, coordinated action on these constraints.

## Figures and Tables

**Table 2:** Serological and molecular tests used in the diagnosis of maternal and congenital syphilis by population.

Test	Population	Detects	Approx. Performance	Clinical Use	Ref.
Maternal screening and diagnosis					
RPR	Maternal	Reagin (anti-cardiolipin) antibodies, not the organism	Sensitivity ≈ 86%; specificity ≈ 94% (stage-dependent; lower in early primary and some late latent infection)	Screening; quantitative titre for treatment monitoring	[24,33]
VDRL	Maternal	Reagin (anti-cardiolipin) antibodies	Comparable to RPR; maternal titre ≥ 1:32 predicted CS (93% sensitivity, 78% specificity)	Screening and monitoring; required for CSF testing in suspected neurosyphilis	[33,34]
TPPA/TPHA	Maternal	Treponemal antibodies (agglutination)	High specificity; confirmatory standard	Confirms a reactive non-treponemal screen (traditional algorithm); remains reactive for life	[8]
FTA-ABS	Maternal	Treponemal antibodies (immunofluorescence)	Sensitivity ≈ 84% (primary) to ≈100% (later stages); specificity ≈ 96%	Confirmatory testing	[33]
EIA/CLIA	Maternal	Treponemal IgG/IgM (automated)	Sensitivity ≈ 99%; specificity ≈ 83–99% (assay-dependent)	Initial screen in the reverse algorithm; high-volume laboratories	[8,33]
PCR/NAAT	Maternal	Treponemal DNA	High specificity when positive; sensitivity depends on specimen	Direct detection in lesion exudate; reference and research use	[35]
Neonatal diagnosis					
Quantitative RPR/VDRL (infant serum)	Neonatal	Reagin antibodies	Interpreted against the maternal titre: an infant titre ≥ 4-fold higher supports infection	Main serological test in neonates; read with maternal titre, examination and maternal treatment history	[33]
Treponemal tests (TPPA, FTA-ABS, EIA/CLIA) on infant serum	Neonatal	Treponemal IgG, mostly maternal in origin	Not applicable	Not recommended for diagnosis: maternal IgG crosses the placenta and can persist up to about 15 months	[33]
PCR/NAAT; placental histology	Neonatal	Treponemal DNA; villitis, obliterative endarteritis	High specificity when positive; sensitivity depends on specimen	Corroborates infection in equivocal cases; largely confined to reference and research laboratories in South Africa	[10,35]
Point-of-care treponemal IgM assays	Neonatal	Treponemal IgM	Early studies show promising discrimination between risk categories	Investigational; not part of routine algorithms	[13,33]

RPR, rapid plasma reagin; VDRL, Venereal Disease Research Laboratory; TPPA, Treponema pallidum particle agglutination; TPHA, T. pallidum haemagglutination; FTA-ABS, fluorescent treponemal antibody absorption; EIA, enzyme immunoassay; CLIA, chemiluminescence immunoassay; PCR, polymerase chain reaction; NAAT, nucleic acid amplification test; CSF, cerebrospinal fluid; CS, congenital syphilis. Performance estimates vary by disease stage, assay and population. Neonatal diagnosis relies on a structured risk assessment (Figure 2) rather than any single test.

**Table 3:** Strategic actions required for elimination of CS in South Africa, by health system level.

Level	Strategic Actions	Reference
Clinical	Test for syphilis at least four times in pregnancy, as per the 2023 guideline—at booking (before 20 weeks), at least twice in the third trimester, and at delivery—particularly for women with no or late antenatal care Treat promptly on a reactive screen with benzathine penicillin G (BPG) appropriate to the stage of infection, and retest to confirm response Apply the structured neonatal risk classification consistently (Figure 2)	[14,24,33]
Facility	Strengthen antenatal appointment scheduling and defaulter tracing Keep a local BPG buffer stock and monitor stock levels routinely Integrate syphilis testing and follow-up into pre-exposure prophylaxis (PrEP) and antiretroviral therapy (ART) visits Notify and treat sexual partners	[26,44,45,52]
District	Prioritise districts using notification rates together with cascade indicators (late booking, treatment completion, retesting) and surveillance completeness, as low notification may reflect under-reporting Conduct facility-based case-finding audits and recurrent health worker training Provide district dashboards with feedback to facilities	[14,24,47]
National	Secure the BPG supply chain with a national buffer stock and interim shortage guidance: prioritise stock for pregnant women and infants, and use alternative antibiotics only under specialist guidance, as their evidence in pregnancy is limited Enforce the 2023 testing guideline Pursue an intermediate WHO Path to Elimination tier (Bronze, then Silver) and align programme metrics with the WHO validation domains: data systems, laboratory capacity, programme performance, and human rights, gender equality and community engagement	[9,24,26,45,52,58,59]

BPG, benzathine penicillin G; PrEP, pre-exposure prophylaxis; ART, antiretroviral therapy; WHO, World Health Organization. Actions are cumulative across levels and depend on each other; for example, secure BPG supply at national level supports treatment completion at facility level.

## Data Availability

No new data were created in this study. Data supporting the reported findings are available in the cited references.
